# *Castanea sativa* Mill. Flowers amongst the Most Powerful Antioxidant Matrices: A Phytochemical Approach in Decoctions and Infusions

**DOI:** 10.1155/2014/232956

**Published:** 2014-04-14

**Authors:** Márcio Carocho, Lillian Barros, Albino Bento, Celestino Santos-Buelga, Patricia Morales, Isabel C. F. R. Ferreira

**Affiliations:** ^1^Mountain Research Center (CIMO), ESA, Polytechnic Institute of Bragança, Campus de Santa Apolónia 1172, 5301-855 Bragança, Portugal; ^2^Department of Nutrition and Bromatology II, Faculty of Pharmacy, Complutense University of Madrid, Pza Ramón y Cajal, s/n., 28040 Madrid, Spain; ^3^Grupo de Investigación en Polifenoles (GIP), Faculty of Pharmacy, University of Salamanca, Campus Miguel de Unamuno, 37007 Salamanca, Spain

## Abstract

Infusions and decoction of chestnut tree flowers have been used for different medical purposes, but their phytochemical profile and antioxidant activity are still mostly unknown. Herein, decoctions and infusions of flowers from the two most appreciated chestnut cultivars (*longal* and *judia*) in Trás-os-Montes, Portugal, were prepared and characterized with regard to their content in free sugars, organic acids, and phenolic compounds, such as flavonoids and hydrolyzable tannins, and their antioxidant activity. Overall, the decoction of the cultivar *judia* was the sample with both the highest quantity of flavonoids and antioxidant activity. The phenolic compound with the highest abundance in all samples was trigalloyl-HHDP-glucoside, followed by pentagalloyl glucoside. The sample with the highest quantity of total phenolic compounds was *judia* infusion, closely followed by *longal* decoction, which also gave the highest quantities of ellagitannins. Regarding sugars and organic acids, the profiles were more similar. These results corroborate ancestral claims of the health benefits of infusions and decoctions of chestnut flowers.

## 1. Introduction

In the Trás-os-Montes region of Portugal and across a good part of the Mediterranean countries, chestnut trees are a considerable part of the landscape. These trees and their respective nuts have been important in the past and are still a source of income for those regions. In Portugal, chestnuts are almost totally exported, translating into a revenue of 32 million euros in 2012 [1, 2]. The chestnut tree has a variety of applications; the nuts are used for human and animal feed, being widely appreciated and even transformed into many typical dishes and desserts. The wood is used for high class furniture. The leaves are used in many ethnobotanic formulations against colds, coughs, diarrhea, and even high blood cholesterol [3]. Furthermore, some patents indicate the use of chestnut flowers in beverages like teas and refreshments [4–6].

The nutritional and bioactive properties of the fruits and flowers of chestnuts have been reported [7–9]. Barros et al. [8, 9] described the high antioxidant potential and phenolic compounds profile of methanolic extracts obtained from the flowers. In fact, some of the most antioxidant molecules are not always found in fruits, but in richer polyphenolic matrices like flowers. Other antioxidant molecules include some organic acids that among many other beneficial activities are known to act against free radicals [8, 10].

The characterization of antioxidant molecules present in flowers is fundamental to draw conclusions concerning their antioxidant potential, which could be interesting for the food industry, by adding antioxidant extracts to foodstuffs or using them in coatings, in order to extend their shelf-life and reduce consumption of chemical additives [11].

In this report, the decoctions and infusions of flowers from the two most appreciated chestnut cultivars (*longal* and* judia*) from Trás-os-Montes, Portugal, were characterized regarding their content of hydrophilic antioxidant molecules (free sugars, organic acids, and phenolic compounds), reducing power, free radicals scavenging activity, and lipid peroxidation inhibition.

## 2. Materials and Methods

### 2.1. Standards and Reagents

Acetonitrile 99.9%, of high performance liquid chromatography (HPLC) grade, and sulphuric acid were acquired from Fisher Scientific (Lisbon, Portugal). Formic acid was acquired from Panreac (Barcelona, Spain). Sugar standards [D(−)-fructose, D(+)-glucose anhydrous and D(+)-sucrose], organic acid standards (malic acid, shikinic acid; oxalic acid and quinic acid), and trolox (6-hydroxy-2,5,7,8-tetramethylchroman-2-carboxylic acid) were acquired from Sigma Chemical Co. (Saint Louis, MO, USA). Phenolic compound standards (catechin, gallic acid, isorhamnetin 3-*O*-glucoside, kaempferol 3-*O*-glucoside, kaempferol 3-*O*-rutinoside, myricetin, quercetin 3-*O*-glucoside and quercetin 3-*O*-rutinoside) were purchased from Extrasynthese (Genay, France). 2,2-Diphenyl-1-picrylhydrazyl (DPPH) was obtained from Alfa Aesar (Ward Hill, MA, USA). Water was treated by means of a Milli-Q water purification system (TGI Pure Water Systems, Greenville, SC, USA).

### 2.2. Flower Samples

*Castanea sativa *Mill. flowers of the cultivars* judia* and* longal* were collected in June 2013 in Oleiros, Bragança (north-eastern Portugal) (41°51′02′′N, 6°49′54′′W). The specimens were lyophilized (FreeZone 4.5, Labconco, Kansas, USA), milled down to a fine powder, and finally stored at −5°C until analysis.

### 2.3. Preparation of the Decoctions and Infusions

For the infusions preparation, the lyophilized flowers (1 g) were added to 200 mL of boiling distilled water, left to stand for 5 min, and finally filtered through a Whatman filter paper. The obtained infusions were frozen and lyophilized.

For the decoctions preparation, the lyophilized flowers (1 g) were added to 200 mL of boiling distilled water, boiled for 5 min, and then left to stand at room temperature for 5 more minutes. After filtration through a Whatman filter paper, the obtained decoctions were frozen and lyophilized.

### 2.4. Analysis of Free Sugars

Free sugars were determined by HPLC coupled to a refraction index (RI) detector as described previously [8]. The equipment consisted of a pump (Knauer, Smartline System 1000, Berlin, Germany), a degasser (Smartline Manager 5000), an autosampler (AS-2057 Jasco, Easton, MD, USA), and a RI detector (Knauer Smartline 2300). The identification was achieved by comparing the relative retention times of sample peaks with standards. Quantification was made by the internal standard method, and the results are expressed in mg per g of lyophilized decoction or infusion.

### 2.5. Analysis of Organic Acids

Organic acids were determined following a procedure previously optimized and described by the authors [10]. Analysis was performed on a Shimadzu 20A series ultra-fast liquid chromatograph (UFLC, Shimadzu Cooperation, Kyoto, Japan) coupled to photodiode array detector (PDA, Shimadzu), using 215 nm and 245 nm as the preferred wavelengths. Separation was achieved on a SphereClone (Phenomenex, Torrance, CA, USA) reverse phase C18 column (5 *μ*m, 250 mm × 4.6 mm i.d) thermostatted at 35°C. Analytes were eluted with 3.6 mM sulphuric acid at a flow-rate of 0.8 mL/min. The organic acids found were quantified by comparison of the area of their peaks recorded at 215 nm or 245 nm (for ascorbic acid) with calibration curves obtained from commercial standards of each compound: oxalic acid (*y* = 1*x*107*x* + 96178; *R* ^2^ = 0.999); quinic acid (*y* = 601768*x* + 8853.2; *R* ^2^ = 1); malic acid (*y* = 952269*x* + 17803; *R* ^2^ = 1); shikimic acid (*y* = 8*x*107 + 55079; *R* ^2^ = 0.999). The results were expressed in mg per g of lyophilized decoction or infusion.

### 2.6. Analysis of Phenolic Compounds

Phenolic compounds were determined by HPLC (Hewlett-Packard 1100, Agilent Technologies, Santa Clara, CA, USA) as previously described [9]. Double online detection was carried out with a PDA using 280 nm and 370 nm as the preferred wavelengths and a mass spectrometer (MS) connected to the HPLC system via the PDA cell outlet. Mass spectrometric detection was performed by means of an API 3200 (Applied Biosystems, Darmstadt, Germany) triple quadrupole-ion trap analyser equipped with an ESI source. Spectra were recorded in negative ion mode between* m/z* 100 and 1700. The phenolic compounds were characterized according to their UV, mass spectra, retention times, and comparison with authentic standards when available. For the quantitative analysis of phenolic compounds, a baseline to valley integration with baseline projection mode was used to calculate peak areas. For quantification, calibration curves were generated by injection of known concentration (2.5–100 *μ*g/mL) of standard compounds: catechin (*y* = 132.76*x* − 59.658; *R* ^2^ = 1); gallic acid (*y* = 556.94*x* − 738.37; *R* ^2^ = 0.999); isorhamnetin-3-*O*-glucoside (*y* = 262.31*x* − 9.8958; *R* ^2^ = 1); kaempferol-3-*O*-glucoside (*y* = 190.75*x* − 36.158; *R* ^2^ = 1); kaempferol-3-*O*-rutinoside (*y* = 175.02*x* − 43.877; *R* ^2^ = 0.999); myricetin (*y* = 778*x* − 1454.3; *R* ^2^ = 0.999); quercetin-3-*O*-glucoside (*y* = 316.48*x* − 2.9142; *R* ^2^ = 1.000); and quercetin-3-*O*-rutinoside (*y* = 222.79*x* − 243.11; *R* ^2^ = 0.999). The results were expressed in mg per g of lyophilized decoction or infusion.

### 2.7. Evaluation of Antioxidant Activity

The* in vitro* antioxidant activity assays were performed following the previously described methodology of Barros et al. [8]. The lyophilized infusions and decoctions were dissolved in water (final concentration 10 mg/mL); the final solution was further diluted to different concentrations to be used in the following assays. DPPH radical-scavenging activity was evaluated using an ELX800 microplate Reader (Bio-Tek Instruments, Inc.; Winooski, VT, USA) and calculated as a percentage of DPPH discolouration after 1 hour of incubation with the antioxidant extract, using the formula: [(*A*_DPPH_ − *A*_*S*_)/*A*_DPPH_]  × 100, where *A*_*S*_ is the absorbance of the solution containing the sample at 515 nm, and *A*_DPPH_ is the absorbance of the DPPH solution. Reducing power was evaluated by the capacity to reduce Fe^3+^ into Fe^2+^, measuring the absorbance at 690 nm in the microplate reader mentioned. Inhibition of *β*-carotene bleaching was evaluated through the *β*-carotene/linoleate assay; the neutralization of linoleate free radicals avoids *β*-carotene bleaching, which is measured by the formula: (*β*-carotene absorbance after 2 h of assay/initial absorbance) × 100. Lipid peroxidation inhibition in porcine (*Sus scrofa*) brain homogenates was evaluated by the decrease in thiobarbituric acid reactive substances (TBARS); the colour intensity of the malondialdehyde-thiobarbituric acid (MDA-TBA) was measured by its absorbance at 532 nm; the inhibition ratio (%) was calculated using the following formula: [(*A* − *B*)/*A*] × 100%, where *A* and *B* were the absorbance of the control and the sample solution, respectively. The results of the antioxidant activity were expressed in EC_50_ value (sample concentration providing 50% of antioxidant activity or 0.5 of absorbance in the reducing power assay).

### 2.8. Statistical Analysis

All the assays were carried out in triplicate for both decoctions and infusions of each cultivar. The results are expressed as mean values ± standard deviations (SD). The statistical differences represented by letters were obtained through one-way analysis of variance (ANOVA) followed by Tukey's honestly significant difference post hoc test with *α* = 0.05. Statistical analyses were carried out using the SPSS v. 18.0 program.

## 3. Results and Discussion

### 3.1. Analysis of Free Sugars and Organic Acids

Three free sugars, namely, fructose, glucose, and sucrose, were detected in all the samples (Table 1). Sucrose was the least abundant sugar, while fructose and glucose were the most abundant in the cultivars* judia* and* longal*, respectively. This had been also reported for methanolic extracts of* C. sativa* flowers [9]. Glucose and fructose were present at higher levels in decoctions than infusions, probably due to the longer extraction time, albeit significant differences were only found in the case of the cultivar* longal*. By contrast, higher concentrations of sucrose were extracted in the infusions, although the increase was only statistically significant in the case of* judia. *No significant differences were observed among samples.

Oxalic, quinic, malic, and shikimic acids were quantified in all the samples, with quinic acid being the most abundant acid (Table 1). Decoctions generally yielded higher quantities of oxalic acid in comparison to infusions, and statistical differences for this compound were only noted for the infusion of* longal*. The* longal* cultivar did not display statistical differences for quinic acid in both extraction methods, but for* judia* statistical differences were found between extraction methods. Malic acid and shikimic acid displayed similar concentrations in all the samples, whereas some differences were found among samples regarding quinic acid. The decoction of* judia* displayed the highest concentrations of total organic acids of the samples analysed.

### 3.2. Analysis of Phenolic Compounds

**Figure 1:**
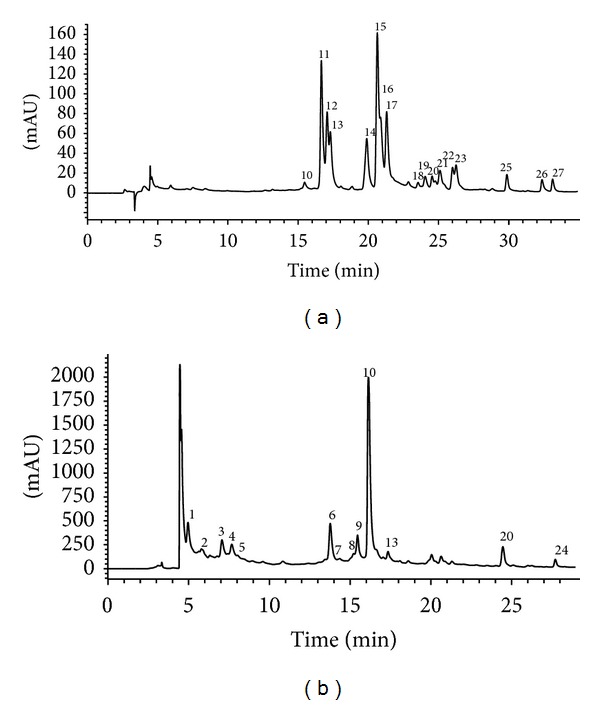
Phenolic profile of the infusion of* Castanea sativa* flowers (*judia* cultivar) recorded at 370 nm (a) and 280 nm (b).

An exemplary phenolic profile of the* judia* cultivar of* C. sativa* recorded at 280 nm and 370 nm is shown in Figure 1. Twenty-seven phenolic compounds were identified in both* judia* and* longal* cultivars (infusion and decoction preparations). Peak characteristics and tentative identities are presented in Table 2.

Peaks 1–4, 6–10, 13, 20, 24, and 25 showed UV spectra coherent with galloyl and hexahydroxydiphenoyl (HHDP) derivatives [12–14]. According to the literature, the main characteristic in the mass spectra of these compounds is the deprotonated molecule [M-H]^−^ and the loss of one or more ellagic acid (302 mu), gallic acid (170 mu), and/or galloyl groups (152 mu) [12, 15, 16]. Peaks 1 and 2 presented a singly charged pseudomolecular ion [M-H]^−^ at* m/z* 783 and daughter ions at* m/z* 481 and 301, which together with their early elution allowed their identification as pedunculagin (i.e., bis-HHDP-glucose) isomers [17]. Mass characteristics of peak 3 ([M-H]^−^ at* m/z* 633; fragment ions at* m/z* 463 and 301) coincided with a galloyl-HHDP-glucose isomer [16, 17], whereas peaks 4, 10, and 13 ([M-H]^−^ at* m/z* 937; fragment ions at* m/z* 767, 637, 467 and 301) were coherent with trigalloyl-HHDP-glucose isomers [13], already reported in* C. sativa* heartwood [18] and flowers [9]. Peak 6 presented a pseudomolecular ion [M-H]^−^ at* m/z* 939, yielding MS^2^ fragment ions at* m/z* 631 [M-2galloyl-3H]^−^, 469 [M-2galloyl-3H-glu]^−^, being identified as pentagalloylglucose. Peak 7 with a pseudomolecular ion [M-H]^−^ at* m/z* 935 and MS^2^ product ions at* m/z* 633 and 301, likely due to the loss of HHDP and galloyl-glucose moieties, was consistent with a galloyl-bis-HHDP-glucose isomer [15, 17, 19]. Pseudomolecular ion ([M-H]^−^ at* m/z* 933) and fragmentation pattern (ions at* m/z* 915, 631, 451 and 301) of peak 8 were in agreement with those attributed to castalagin or vescalagin isomers [17, 20], already reported in* C. sativa* heartwood [18]. Peaks 9, 20, and 24 are most likely galloyl-HHDP derivatives, with an unusual parent ion [M-H]^−^ at* m/z* 907, but with characteristic fragments of this type of compounds (*m/z* at 767, 467 and 169). No structure could be assigned to these peaks that remain unidentified. A fragment ion with* m/z* at 907 was reported as released from the cleavage of a di(HHDP-galloylglucose)-pentose found in pomegranate juice [21] and from unknown ellagitannins present in blackberries [17]. Peak 25 presented an ellagic acid-like UV spectrum (*λ*_max⁡_ around 250 and 368 nm) and a pseudo molecular ion [M-H]^−^ at* m/z* 343, releasing three fragments at* m/z* 328, 313 and 298 mu, corresponding to the successive losses of three methyl groups (−15 mu), which allowed its tentative identification as a tri-*O*-methylellagic acid.

Regarding flavonoids, flavonol derivatives were the main compounds found in the analyzed samples (Table 2). Catechin (peak 5), myricetin 3-*O*-glucoside (peak 12), quercetin 3-*O*-rutinoside (peak 14), quercetin 3-*O*-glucoside (peak 16), kaempferol 3-*O*-rutinoside (peak 18), and kaempferol 3-*O*-glucoside (peak 21) were positively identified according to their retention, mass, and UV-vis characteristics by comparison with commercial standards. Peak 11 was assigned to a myricetin* O*-glucuronide, according to the pseudomolecular ion [M-H]^−^ at* m/z* 497 and MS^2^ fragment released at* m/z* 317 ([M-H-176]^−^, loss of glucuronyl moiety). Peaks 15, 17, 19, and 26 presented UV spectra with *λ*_max⁡_ around 350 nm and an MS^2^ product ion at* m/z* 301 indicating that they correspond to quercetin derivatives. According to their pseudomolecular ions, they were identified as quercetin 3-*O*-glucuronide (peak 15; [M-H]^−^ at* m/z* 477), which was confirmed by comparison with a standard obtained in our laboratory [22], quercetin* O*-hexoside (peak 17; [M-H]^−^ at* m/z* 463), quercetin* O*-pentoside (peak 19; [M-H]^−^ at* m/z* 433), and quercetin* O*-rhamnosyl hexoside (peak 26; [M-H]^−^ at* m/z* 609). Similarly reasoning also allowed assigning peaks 22 and 23 as isorhamnetin* O*-hexoside and isorhamnetin* O*-glucuronide, respectively. Peak 27 should correspond to isorhamnetin* O*-acetylhexoside according to the pseudomolecular ion [M-H]^−^ at* m/z* 519 and MS^2^ fragment released at* m/z* 315 ([M-H-42-162]^−^, loss of an acetylhexoside moiety). The individual polyphenol with the highest concentration in all samples was a trigalloyl-HHDP-glucoside (peak 10), followed by pentagalloyl glucose (peak 6), whereas quercetin 3-*O*-glucuronide (peak 15) and a quercetin hexoside (peak 17) were the most abundant flavonoids in* judia* and* longal* cultivars, respectively (Table 3). The sample with the highest concentration of total polyphenols was the infusion of* judia*, closely followed by the decoction of* longal*. The preparations of the cultivar* judia *presented higher flavonoid levels, while those of* longal* displayed higher concentrations of hydrolyzable tannins (Table 3). The compounds present in the samples were, to some extent, different from those found in a* C. sativa* hydromethanolic extract [9]. Nevertheless, the main ellagitannins found in this study were in accordance with the mentioned study.

### 3.3. Antioxidant Activity

The antioxidant activity of the* C. sativa* samples was determined through various assays, namely, DPPH scavenging activity, reducing power through Prussian-blue assay, inhibition of *β*-carotene bleaching, and finally inhibition of TBARS formation in brain cell homogenates (Table 4). Decoctions showed greater antioxidant activity than infusions; this might be explained by the longer time at boiling point that decoctions were subjected to during extraction. In terms of scavenging of DPPH radicals, statistically significant differences were found between decoctions and infusions. Reducing power, *β*-carotene bleaching inhibition and TBARS assays gave statistically significant different results among samples, but the lowest EC_50_ values were always obtained in the TBARS assay, this could be due to its specificity, sensibility, and low quantity of interferences.

This assay was the only one that reported lower EC_50_ values for infusions, which can be related to the low heat resistance of antioxidants that inhibit lipid peroxidation, like tocopherols and other vitamins [23].

Among the four samples, the decoction of* judia* proved to be the most antioxidant among the samples analysed. This could be explained by antioxidant variability of cultivars, translating into a higher antioxidant potential of* judia* when compared to* longal*. Further research should be carried out to determine what antioxidants apart from phenolic compounds are present in these flowers that could help clarify this antioxidant variability between cultivars. Nevertheless, in general, all the studied samples proved to be powerful antioxidants when compared to other herbal matrices previously studied by the authors [8, 9].

## 4. Conclusion

The ancestral claims of health benefits of chestnuts flowers were partially corroborated in this paper. The organic acids could be responsible for some antioxidant potential, but further research can be carried out in terms on tocopherols and other vitamins and minerals that could provide more insight into the total amount of antioxidants. Further research should be carried out on the potentiality of these flowers in the pharmaceutical and food industries, among others. Pharmaceutical industries could use the flowers as excipients for dietary supplements, benefiting from their natural content in polyphenols for health purposes. The food industry, which has recently started to intensify its search for natural conservatives and additives, to add to transformed foodstuffs, meeting the consumers habits, can use the high antioxidant power and natural high abundance of tannins in the flowers to preserve food and inhibit lipid deterioration and microorganism development. Although quite beneficial, the flowers used for these purposes should not be picked from the tree, but rather from the ground to avoid interference of pollination. Methanolic extractions have also been performed for chestnut flowers and yielded good results [8, 9], although the usage of methanol is not recommended in the food industry; but on the other hand, extractions with this solvent could carry hydrophobic antioxidant compounds.

## Figures and Tables

**Table 1:** Content of free sugars and organic acids in mg/g of lyophilized decoction/infusion of the flowers of two *Castanea sativa* cultivars.

	Decoction *judia *	Decoction *longal *	Infusion *judia *	Infusion *longal *
Sugars				
Fructose	160.41 ± 0.01^a^	152.08 ± 6.52^a^	148.94 ± 4.60^a^	123.58 ± 1.76^b^
Glucose	149.09 ± 0.04^b^	191.91 ± 7.35^a^	145.71 ± 5.63^b^	164.07 ± 2.31^b^
Sucrose	27.01 ± 0.45^b^	25.69 ± 0.99^b^	35.68 ± 1.45^a^	26.67 ± 0.50^b^

Total	336.51 ± 0.48^a^	369.69 ± 14.85^a^	330.32 ± 11.69^a^	314.32 ± 4.57^a^

Organic Acids				
Oxalic acid	72.91 ± 1.82^a^	71.85 ± 0.52^ab^	43.42 ± 0.75^c^	55.84 ± 1.82^bc^
Quinic acid	84.61 ± 0.64^a^	52.59 ± 3.14^b^	69.04 ± 2.81^ab^	61.63 ± 4.40^b^
Malic acid	27.17 ± 1.36^a^	22.94 ± 0.10^a^	25.03 ± 0.52^a^	20.82 ± 0.70^a^
Shikimic acid	1.83 ± 0.02^a^	1.26 ± 0.08^a^	1.35 ± 0.02^a^	1.35 ± 0.07^a^

Total	186.52 ± 3.84^a^	148.65 ± 2.79^ab^	138.83 ± 2.61^b^	139.64 ± 6.99^b^

In each row different letters mean significant differences with a *P* < 0.05. Letters refer to Tukey's post hoc test; therefore, significant different values were classified using letters in alphabetic order.

**Table 2:** Retention time (Rt), wavelength of maximum absorption (*λ*_max⁡_), mass spectral data, and tentative identification of phenolic compounds in decoctions and infusions of *Castanea sativa* flowers.

Peak	Rt (min)	*λ*_max⁡_ (nm)	Pseudomolecular ion [M-H]^−^ (*m/z*)	MS^2^ (*m/z*) (% of base peak)	Tentative identification
1	4.96	276	783	481 (10), 301 (41)	Pedunculagin isomer (bis-HHDP-glucose)
2	5.79	268	783	481 (6), 301 (45)	Pedunculagin isomer (bis-HHDP-glucose)
3	7.06	272	633	463 (17), 301 (100)	Galloyl-HHDP-glucose
4	7.67	274	937	637 (15), 467 (2), 301 (4)	Trigalloyl-HHDP-glucose
5	8.02	278	289	245 (91), 203 (60), 137 (38)	(+)-Catechin
6	13.77	276	939	631 (31), 469 (66), 169 (100)	Pentagalloyl glucose
7	14.92	276	935	633 (15), 301 (18)	Galloyl-bis-HHDP-glucose
8	15.25	274	933	915 (5), 633 (8), 451 (24), 301 (7)	Castalagin/vescalagin
9	15.46	278	907	767 (3), 607 (24), 467 (35), 169 (5)	Galloyl-HHDP derivative
10	16.13	274	937	767 (2), 637 (8), 467 (68), 301 (10)	Trigalloyl-HHDP-glucoside
11	16.65	358	493	317 (100)	Myricetin *O*-glucuronide
12	17.07	350	479	317 (100)	Myricetin 3-*O*-glucoside
13	17.30	274	937	767 (2), 637 (5), 467 (58), 301 (7)	Trigalloyl-HHDP-glucoside
14	19.89	356	609	301 (100)	Quecetin 3-*O*-rutinoside
15	20.64	356	477	301 (100)	Quercetin 3-*O*-glucuronide
16	20.86	356	463	301 (100)	Quercetin 3-*O*-glucoside
17	21.30	356	463	301 (100)	Quercetin *O*-hexoside
18	23.54	350	593	285 (100)	Kaempferol 3-*O*-rutinoside
19	24.05	354	433	301 (100)	Quercetin *O*-pentoside
20	24.46	268	907	767 (3), 607 (23), 467 (67), 169 (7)	Galloyl-HHDP derivative
21	25.11	348	477	285 (100)	Kaempferol 3-*O*-glucoside
22	26.00	354	477	315 (100)	Isorhamnetin *O*-hexoside
23	26.25	354	491	315 (100)	Isorhamnetin *O*-glucuronide
24	27.71	274	907	767 (2), 607 (27), 467 (76), 169 (8)	Galloyl-HHDP derivative
25	29.87	250/368	343	328 (97), 313 (100), 298 (36)	Tri-*O*-methylellagic acid
26	32.38	358	609	463 (76), 301 (40)	Quercetin *O*-rhamnosyl hexoside
27	33.13	356	519	477 (5), 315 (77)	Isorhamnetin *O*-acetylhexoside

**Table 3:** Quantification of phenolic compounds in infusions and decoctions of *Castanea sativa* flowers expressed in mg/g of lyophilized decoction/infusion.

Compounds	Decoction *judia *	Decoction *longal *	Infusion *judia *	Infusion *longal *
Pedunculagin isomer (bis-HHDP-glucose)	5.21 ± 0.11	8.48 ± 0.51	5.98 ± 0.16	7.68 ± 0.11
Pedunculagin isomer (bis-HHDP-glucose)	1.12 ± 0.09	1.44 ± 0.19	0.92 ± 0.92	1.21 ± 0.04
Galloyl-HHDP-glucose	3.07 ± 0.14	3.18 ± 0.00	3.18 ± 0.07	3.05 ± 0.09
Trigalloyl-HHDP-glucose	2.81 ± 0.25	5.51 ± 0.06	2.60 ± 0.06	4.30 ± 0.47
(+)-Catechin	1.14 ± 0.12	1.58 ± 0.14	1.11 ± 0.03	1.10 ± 0.05
Pentagalloyl glucose	5.61 ± 0.08	6.04 ± 0.15	5.73 ± 0.14	6.47 ± 0.15
Galloyl-bis-HHDP-glucose	0.45 ± 0.04	0.29 ± 0.07	0.66 ± 0.01	0.30 ± 0.01
Castalagin/vescalagin	0.75 ± 0.06	0.73 ± 0.11	1.06 ± 0.09	1.61 ± 0.04
Galloyl-HHDP derivative	0.38 ± 0.02	0.55 ± 0.07	3.60 ± 0.16	3.72 ± 0.01
Trigalloyl-HHDP-glucoside	26.72 ± 0.15	28.16 ± 0.11	30.70 ± 0.40	28.73 ± 1.34
Myricetin *O*-glucuronide	1.13 ± 0.02	0.09 ± 0.00	0.96 ± 0.01	0.08 ± 0.00
Myricetin 3-*O*-glucoside	0.66 ± 0.01	0.09 ± 0.01	0.60 ± 0.01	0.09 ± 0.00
Trigalloyl-HHDP-glucoside	1.33 ± 0.00	0.54 ± 0.01	1.61 ± 0.05	0.70 ± 0.04
Quecetin 3-*O*-rutinoside	1.57 ± 0.01	0.52 ± 0.03	1.31 ± 0.10	0.48 ± 0.05
Quercetin 3-*O*-glucuronide	3.55 ± 0.10	1.56 ± 0.04	3.38 ± 0.01	1.49 ± 0.07
Quercetin 3-O-glucoside*	1.80 ± 0.01	0.89 ± 0.02	1.60 ± 0.04	0.91 ± 0.10
Quercetin *O*-hexoside	2.21 ± 0.07	1.82 ± 0.05	2.11 ± 0.02	1.85 ± 0.06
Kaempferol 3-*O*-rutinoside	0.21 ± 0.01	0.21 ± 0.03	0.18 ± 0.01	0.21 ± 0.01
Quercetin *O*-pentoside	0.43 ± 0.02	0.20 ± 0.01	0.34 ± 0.00	0.22 ± 0.02
Galloyl-HHDP derivative	4.18 ± 0.10	4.54 ± 0.14	2.41 ± 0.14	1.96 ± 0.12
Kaempferol 3-*O*-glucoside	0.53 ± 0.00	0.49 ± 0.01	0.41 ± 0.00	0.46 ± 0.04
Isorhamnetin *O*-hexoside	0.33 ± 0.04	0.27 ± 0.01	0.27 ± 0.00	0.30 ± 0.01
Isorhamnetin *O*-glucuronide	0.35 ± 0.03	0.50 ± 0.02	0.33 ± 0.01	0.46 ± 0.04
Galloyl-HHDP derivative	2.04 ± 0.06	2.02 ± 0.03	0.72 ± 0.01	0.59 ± 0.01
Tri-*O*-methylellagic acid	0.10 ± 0.01	tr	0.08 ± 0.01	tr
Quercetin *O*-rhamnosyl hexoside	0.23 ± 0.00	0.10 ± 0.01	0.23 ± 0.03	0.09 ± 0.00
Isorhamnetin *O*-acetylhexoside	0.11 ± 0.00	0.08 ± 0.01	0.12 ± 0.01	0.04 ± 0.00

Total flavonoids	**14.26 ± 0.14** ^ a^	**8.38 ± 0.01** ^ c^	**12.94 ± 0.17** ^ b^	**7.79 ± 0.35** ^ d^
Total hydrolyzable tannins	**53.78 ± 0.31** ^ c^	**61.49 ± 0.80** ^ a^	**59.25 ± 0.03** ^ b^	**60.32 ± 1.87** ^ ab^
Total phenolic compounds	**68.04 ± 0.18** ^ c^	**69.88 ± 0.81** ^ bc^	**72.20 ± 0.14** ^ a^	**68.10 ± 1.52** ^ c^

In each row different letters mean significant differences with a *P* < 0.05. They refer to Tukey's post hoc test; therefore, significant different values were classified using letters in alphabetic order. tr: compound detected in trace amount. *Estimation due to its low resolution.

**Table 4:** Antioxidant activity of decoctions and infusions of the flowers of two *Castanea sativa* cultivars.

Antioxidant activity (EC_50_ values, *μ*g/mL)	Decoction *judia *	Decoction *longal *	Infusion *judia *	Infusion *longal *
DPPH scavenging activity	99.47 ± 0.006^b^	100.04 ± 0.01^b^	126.61 ± 0.005^a^	133.56 ± 0.005^a^
Reducing power	68.51 ± 0.001^d^	76.07 ± 0.001^c^	90.65 ± 0.001^b^	98.79 ± 0.001^a^
*β*-carotene bleaching inhibition	47.89 ± 0.002^d^	184.92 ± 0.001^b^	177.23 ± 0.004^c^	195.10 ± 0.01^a^
TBARS inhibition	38.73 ± 0.001^b^	48.63 ± 0.000^a^	15.24 ± 0.002^d^	19.79 ± 0.003^c^

EC_50_ values correspond to the sample concentration achieving 50% of antioxidant activity or 0.5 of absorbance in the reducing power assay. In each row different letters mean significant differences with a *P* < 0.05. They refer to Tukey's post hoc test; therefore, significant different values were classified using letters in alphabetic order.
